# Metabolomic profiling of preterm birth in pregnant women living with HIV

**DOI:** 10.1007/s11306-023-02055-1

**Published:** 2023-10-25

**Authors:** Nicole H. Tobin, Aisling Murphy, Fan Li, Sean S. Brummel, Mary Glenn Fowler, James A. Mcintyre, Judith S. Currier, Tsungai Chipato, Patricia M. Flynn, Luis A. Gadama, Friday Saidi, Clemensia Nakabiito, Brian J. Koos, Grace M. Aldrovandi, the IMPAACT 1077BF/1077FF PROMISE Study Team

**Affiliations:** 1 Division of Infectious Diseases, Department of Pediatrics, David Geffen School of Medicine at the University of California, Los Angeles, CA USA; 2 Department of Obstetrics and Gynecology, David Geffen School of Medicine at the University of California, Los Angeles, CA USA; 3 Center for Biostatistics in AIDS Research, Harvard T.H. Chan School of Public Health, Boston, MA USA; 4 Department of Pathology, Johns Hopkins University School of Medicine, Baltimore, MD USA; 5 Anova Health Institute, Johannesburg, South Africa; 6 School of Public Health and Family Medicine, University of Cape Town, Cape Town, South Africa; 7 Division of Infectious Diseases, Department of Internal Medicine, David Geffen School of Medicine at the University of California, Los Angeles, CA USA; 8 University of Zimbabwe College of Health Sciences, Harare, Zimbabwe; 9 Department of Infectious Diseases, St. Jude Children’s Research Hospital, Memphis, TN USA; 10 Department of Obstetrics and Gynecology, Johns Hopkins Research Project, Kamuzu University of Health Sciences, Blantyre, Malawi; 11 University of North Carolina Project Malawi, Lilongwe, Malawi; 12 MU-JHU Research Collaboration (MUJHU CARE LTD) CRS, Kampala, Uganda

**Keywords:** Preterm birth, Women living with HIV, Metabolomics, Zidovudine, Plasma, Dried blood spots

## Abstract

**Background:**

Preterm birth is a leading cause of death in children under the age of five. The risk of preterm birth is increased by maternal HIV infection as well as by certain antiretroviral regimens, leading to a disproportionate burden on low- and medium-income settings where HIV is most prevalent. Despite decades of research, the mechanisms underlying spontaneous preterm birth, particularly in resource limited areas with high HIV infection rates, are still poorly understood and accurate prediction and therapeutic intervention remain elusive.

**Objectives:**

Metabolomics was utilized to identify profiles of preterm birth among pregnant women living with HIV on two different antiretroviral therapy (ART) regimens.

**Methods:**

This pilot study comprised 100 mother-infant dyads prior to antiretroviral initiation, on zidovudine monotherapy or on protease inhibitor-based antiretroviral therapy. Pregnancies that resulted in preterm births were matched 1:1 with controls by gestational age at time of sample collection. Maternal plasma and blood spots at 23–35 weeks gestation and infant dried blood spots at birth, were assayed using an untargeted metabolomics method. Linear regression and random forests classification models were used to identify shared and treatment-specific markers of preterm birth.

**Results:**

Classification models for preterm birth achieved accuracies of 95.5%, 95.7%, and 80.7% in the untreated, zidovudine monotherapy, and protease inhibitor-based treatment groups, respectively. Urate, methionine sulfone, cortisone, and 17α-hydroxypregnanolone glucuronide were identified as shared markers of preterm birth. Other compounds including hippurate and *N*-acetyl-1-methylhistidine were found to be significantly altered in a treatment-specific context.

**Conclusion:**

This study identified previously known as well as novel metabolomic features of preterm birth in pregnant women living with HIV. Validation of these models in a larger, independent cohort is necessary to ascertain whether they can be utilized to predict preterm birth during a stage of gestation that allows for therapeutic intervention or more effective resource allocation.

**Supplementary Information:**

The online version contains supplementary material available at 10.1007/s11306-023-02055-1.

## Introduction

Prematurity or preterm birth (PTB) is defined as birth before 37 weeks of gestation and is the leading cause of death in children under the age of five. An estimated 15 million infants are born preterm and approximately one million children die of complications of PTB annually (World Health Organization, 2020). The majority of PTB occur spontaneously as opposed to iatrogenic causes. Despite its tremendous burden on public health, spontaneous PTB (sPTB) remains poorly understood and relatively difficult to predict or prevent (Esplin et al., 2017; Honest et al., 2012). In fact, only recently has spontaneous PTB been considered as an obstetric ‘syndrome’, wherein one or more of a multitude of possible upstream pathways ultimately lead to a final common presentation (Souza et al., 2019a). Some of the implicated etiological pathways involve inflammation, decidual hemorrhage, immunological factors, uterine stretch and endocrine derangement (Manuck et al., 2015; Behrman & Butler, 2007). Progesterone, and to a lesser extent, estrogen appear to have some role. Progesterone supplementation has been used for more than a decade to prevent PTB in at-risk women, although its efficacy has recently been called into question (Blackwell et al., 2020).

HIV infection is associated with an increased rate of PTB, occurring in 15–20% of all pregnancies with notable variation by treatment regimen (Fowler et al., 2016). The etiology of PTB is particularly complex in the context of HIV infection as it may be spontaneous, HIV-associated, antiretroviral therapy (ART)-associated, or a combination thereof. The rate of PTB initially declined with the use of ART as maternal health improved on therapy, but then increased with certain antiretroviral regimens (Powis et al., 2011; Schulte et al., 2007). This increase is particularly notable in women who are receiving protease inhibitors (PI) where rates of PTB increase almost two-fold; in fact, it is estimated that protease inhibitor use may result in 91,000 additional preterm births per year (Fowler et al., 2016; Powis et al., 2011). Over 30% of new HIV infections are estimated to occur in people aged 14–25 years, and young women are substantially more likely to become infected versus men in the same age group (UNAIDS, 2017, 2020). Given the disproportionately high rate of new HIV infections in young women and the higher rates of pregnancy among women living with HIV (WLH) compared with earlier in the epidemic, the number of HIV-associated preterm births is unfortunately likely to increase as well. Therefore, it is of vital importance to better understand the etiology of PTB among this at-risk population.

Accurate prediction of sPTB has proven difficult, particularly in women with no clinically indicated risk or prior history. Recent studies that leverage “omics” technologies to better capture the complex mechanisms underlying spontaneous PTB (Huang et al., 2019; Kindschuh et al., 2023; Lizewska et al., 2018; Morillon et al., 2020; Ngo et al., 2018; Souza et al., 2019b) have shown some promise in identifying markers of sPTB. To the best of our knowledge however, no similar studies have been performed in WLH who are at elevated risk for PTB. Here, we use an untargeted metabolomics approach to identify novel signatures of PTB among pregnant WLH on three different treatment regimens. The goal of this study is to identify common metabolic markers of PTB as well as those specifically altered in the context of different antiretroviral therapies.

## Methods

### Study design and sample collection

A subgroup of 100 pregnant WLH with a CD4 count ≥ 350 cells/mm^3^ or country-specific treatment threshold was selected based on availability of sample aliquots as part of a larger clinical trial (Fowler et al., 2016). Maternal plasma and dried blood spot (DBS) samples were collected between 23 and 35 weeks of gestation either prior to antiretroviral initiation (untreated) or during treatment with either zidovudine monotherapy (ZDV) or a protease-inhibitor based regimen (PI-ART). One woman receiving PI-ART was on tenofovir (TDF) + emtricitabine (FTC) + lopinavir/ritonavir (LPV/r). All other women received ZDV + lamivudine (3TC) + LPV/r. Preterm birth was defined as delivery prior to 37 weeks of gestation, with gestational age assessed by obstetrical estimate due to unavailability of the gold standard ultrasound (Venkatesh et al., 2019). A pediatric assessment of gestational age by newborn examination was also utilized for a sensitivity analysis. The term and preterm groups were matched for treatment group, country of origin, and gestational age at time of sample collection. Infant dried blood spot (DBS) samples were collected from the same 100 mother-infant pairs.

Blood samples were collected in BD Vacutainer tubes with Acid Citrate Dextrose (ACD) and transferred to Whatman 903 Protein Saver Cards. Cards were then air dried for at least four hours and then placed into gas impermeable bags with a desiccant pack and humidity card for long-term storage at − 80 °C. Following DBS preparation, the remaining blood volume was centrifuged at 400×*g* for 10 min. Plasma was then transferred to a new sterile tube and centrifuged again at 800×*g* for 10 min. Aliquots were taken and placed into sterile cryovials for storage at − 80 °C.

### Sample processing and metabolomics

Samples were processed by Metabolon Inc. according to published methods with modifications as described for DBS samples (Evans et al., 2009, 2014; Ford et al., 2020). Untargeted ultra-high-performance liquid chromatography/tandem mass spectrometry of known biochemicals was conducted on plasma and DBS samples by Metabolon Inc. according to published methods (Evans et al., 2009, 2014; Ford et al., 2020). Briefly, aliquots were analyzed using four separate methods: two using acidic positive ion conditions optimized for either more hydrophilic or more hydrophobic conditions, one using basic negative ion optimized conditions, and one using negative ionization conditions. All four methods utilized a Waters ACQUITY ultra-performance liquid chromatography (UPLC) and a Thermo Scientific Q-Exactive high resolution/accurate mass spectrometer interfaced with a heated electrospray ionization (HESI-II) source and Orbitrap mass analyzer operated at 35,000 mass resolution. In the first method, the extract was gradient eluted from a C18 column (Waters UPLC BEH C18-2.1 × 100 mm, 1.7 µm) using water and methanol, containing 0.05% perfluoropentanoic acid (PFPA) and 0.1% formic acid (FA). In the second method, the extract was gradient eluted from the same C18 column using methanol, acetonitrile, water, 0.05% PFPA and 0.01% FA at an overall higher organic content. In the third method, basic extracts were gradient eluted from the column using methanol and water, however with 6.5 mM Ammonium Bicarbonate at pH 8. In the fourth method, elution was performed from a HILIC column (Waters UPLC BEH Amide 2.1 × 150 mm, 1.7 µm) using a gradient consisting of water and acetonitrile with 10 mM Ammonium Formate, pH 10.8. The MS analysis alternated between MS and data-dependent MSn scans using dynamic exclusion. The scan range varied slighted between methods but covered 70–1000 m/z. Additional information regarding UPLC-MS methods are provided in Supplementary File 3.

Missing values were imputed with the minimum quantified value of each biochemical in the sample matrix (plasma or DBS). There were a median of 0 and 2 samples with missing values for plasma and DBS analytes, respectively. All values were then log-transformed and standardized using a Z-transform.

### Statistical analysis

Principal components analysis (PCA) was performed to visualize separation by PTB across the overall metabolite profiles. Permutational multivariate analysis of variance (PERMANOVA) with Euclidean distances, was used to assess PTB, antiretroviral regimen, country of origin, and gestational age at sample as predictors. Linear regression models were built separately with each standardized metabolite value as an outcome using an interaction model with antiretroviral regimen and preterm birth (as assessed by obstetric estimate) as covariates. A sensitivity analysis was also performed with preterm birth defined via newborn examination. Linear regression results were summarized as estimated marginal means.

Random forests classification models were constructed separately for each drug regimen to predict PTB. Based on our prior comparison of DBS and plasma sampling for metabolomics (Tobin et al., 2021), normalized and standardized DBS- and plasma-derived metabolite abundances were used as input for the multi-omics model as well as for separate models within each sample matrix. A power analysis of a random forests classification model specifically in the context of metabolomics data suggests that the current study is sufficiently powered to predict PTB (Acharjee et al., 2020). Country of origin was also included as a covariate due to its significance in the PERMANOVA results. A two-step approach was utilized to select both the optimal number of features as well as the specific features used for each model. In the first step, one hundred forests each comprising 10,000 trees were built to obtain feature importance values calculated as mean decrease in accuracy. In the second step, tenfold cross-validation with a sequentially reduced number of features was then used to identify the optimal number of features to be used. Finally, a sparse model was constructed for each regimen and sample matrix containing the optimal number of metabolites calculated in step 2 (up to a maximum of fifty features to aid interpretability), with features selected by the highest importance as calculated in step 1. Model accuracy, sensitivity, specificity, Matthew’s correlation coefficient (MCC), and area under the receiver operator curve (AUC) were calculated from the out-of-bag error estimate (Breiman, 2001) for the final sparse model. The Benjamini–Hochberg false discovery rate (FDR) method was used to adjust for multiple comparisons and q-values less than 0.05 were accepted as significant.

All analyses were performed in the R statistical environment (version 3.6.3) (R Core Team, 2019). A full list of the R packages used in the analyses is provided in Online Appendix A. Analysis code and data files necessary to reproduce the analyses are available upon request.

## Results

### Cohort demographics

**Fig. 1:**
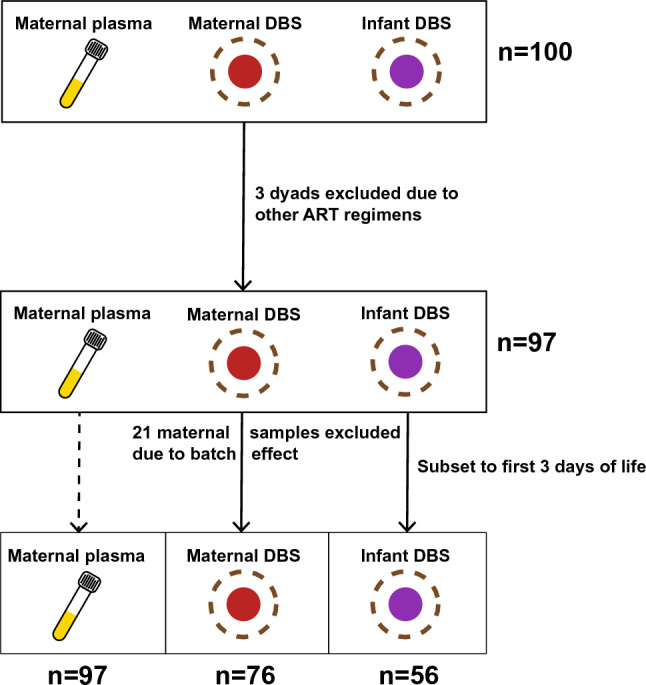
Study design. Schematic showing exclusion criteria and the number of samples used for analysis of maternal plasma, maternal DBS, and infant DBS samples

Untargeted metabolomics was performed on maternal plasma, maternal dried blood spots (DBS), and infant DBS from 100 mother-infant dyads who were either enrolled but yet to be treated (untreated), on ZDV, or on protease inhibitor-based antiretroviral therapy (PI-ART). A strong batch effect was observed among maternal DBS samples from a single study site (see Supplementary Results in Online Resource 1) and therefore the decision was made to exclude these 21 maternal DBS samples, but not their corresponding plasma samples, from all further analyses. Three additional dyads were excluded as they were exposed to other drug regimens during the course of pregnancy. The remaining participants were used for all subsequent analyses of maternal plasma (n = 97, Table 1) and maternal DBS (n = 76, Table S1) samples (Fig. 1).

**Table 1:** Demographic characteristics related to maternal analyses

	Untreated—term^a^	Untreated—preterm^a^	p	ZDV—term^b^	ZDV—preterm^b^	p	PI-ART—term^c^	PI-ART—preterm^c^	p	p—overall
n	15	13		13	18		20	18		
Sex = Male (%)	6 (40.0)	5 (38.5)	1	6 (46.2)	6 (33.3)	0.727	11 (55.0)	8 (44.4)	0.745	0.837
Birthweight (mean (SD), g)	2932.67 (329.20)	2643.08 (498.54)	0.078	2810.91 (481.69)	2583.67 (529.90)	0.273	2829.50 (552.21)	2453.39 (469.96)	**0.031**	0.059
Weight at 1 week (mean (SD), g)	3356.92 (477.13)	2899.75 (408.16)	**0.017**	3073.85 (464.59)	2927.14 (483.89)	0.43	3122.11 (559.79)	2660.56 (676.30)	**0.03**	**0.015**
Gestational age at start of therapy (mean (SD), weeks)	31.00 (2.78)	29.75 (2.84)	0.251	23.48 (4.23)	23.48 (4.20)	0.996	24.76 (2.96)	24.91 (2.90)	0.872	** < 0.001**
Country (%)			0.312			0.879			0.819	0.302
India	1 (6.7)	0 (0.0)		0 ( 0.0)	0 ( 0.0)		1 ( 5.0)	2 (11.1)		
Malawi	8 (53.3)	5 (38.5)		10 (76.9)	13 (72.2)		5 (25.0)	6 (33.3)		
South Africa	6 (40.0)	6 (46.2)		2 (15.4)	4 (22.2)		10 (50.0)	8 (44.4)		
Uganda	0 (0.0)	2 (15.4)		1 ( 7.7)	1 ( 5.6)		3 (15.0)	1 ( 5.6)		
Zambia	0 (0.0)	0 ( 0.0)		0 ( 0.0)	0 ( 0.0)		1 ( 5.0)	1 ( 5.6)		
Gestational age at sample collection (mean (SD), weeks)	31.00 (2.78)	29.75 (2.84)	0.25	30.79 (2.50)	30.83 (2.73)	0.971	30.84 (2.35)	31.23 (2.34)	0.61	0.737
Gestational age at delivery (mean (SD), weeks)	40.58 (3.90)	32.00 (3.10)	** < 0.001**	39.69 (2.44)	33.23 (2.45)	** < 0.001**	39.78 (2.11)	33.55 (1.96)	** < 0.001**	** < 0.001**
Infant age in days (mean (SD))	2.80 (4.83)	3.69 (4.53)	0.62	4.85 (4.83)	17.22 (42.66)	0.309	2.50 (3.59)	2.89 (3.36)	0.733	0.155
Days prior to delivery, plasma (mean (SD))	67.07 (26.21)	15.77 (10.86)	** < 0.001**	62.31 (22.64)	16.83 (10.37)	** < 0.001**	62.60 (19.10)	16.22 (9.48)	** < 0.001**	** < 0.001**
Days prior to delivery, DBS (mean (SD))	67.07 (26.21)	13.62 (7.78)	** < 0.001**	62.31 (22.64)	16.83 (10.37)	** < 0.001**	62.60 (19.10)	16.22 (9.48)	** < 0.001**	** < 0.001**

Bold values indicate p < 0.05

Demographics of the mother-infant dyads used for analysis of maternal plasma samples. P-values are derived from χ^2^ and one-way ANOVA tests for categorical and continuous variables, respectively

^a^Enrolled but had not initiated therapy at the time of sample collection

^b^Zidovudine monotherapy group

^c^Protease inhibitor-based antiretroviral therapy group

### Maternal profiles of preterm birth

**Fig. 2:**
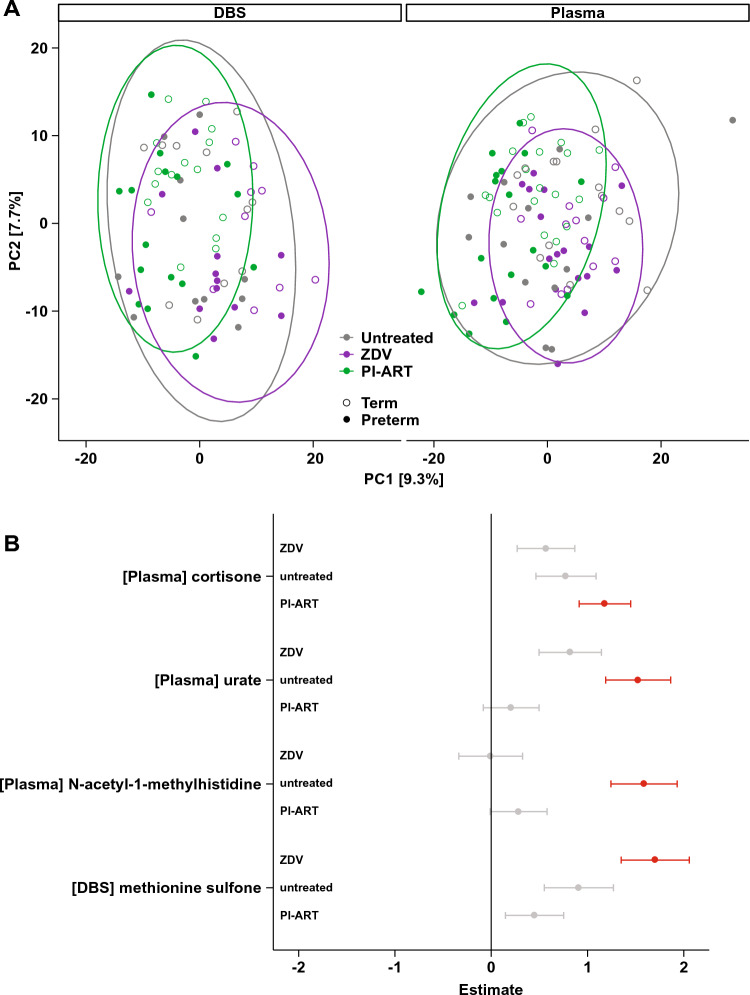
Maternal metabolites in preterm birth. **a** Principal components analysis of maternal metabolite profiles using Euclidean distances. Ellipses show 95% confidence areas for the treatment regimens as marked. Numbers in brackets denote percent of overall variation explained by each component. **b** Coefficients from linear regression analysis of maternal metabolites stratified by treatment regimen. Only metabolites that were significant in any single analysis are shown. Metabolites with positive estimates are increased in women who deliver preterm and metabolites with negative estimates are increased in women who deliver at term. Error bars denote 95% confidence intervals. Values in red are statistically significant with FDR-adjusted p < 0.05

Principal components analysis (PCA) of maternal metabolite profiles revealed weak but statistically significant segregation by PTB, antiretroviral regimen, and country of origin (Fig. 2A and Table S2, PERMANOVA p < 0.001 with 2.0%, 5.2%, and 9.9% of variance explained, respectively). Linear regression revealed 83 statistically significant mean compound differences when comparing women who delivered preterm to those who delivered at term, including methionine sulfone, 17α-hydroxypregnanolone glucuronide, estriol 3-sulfate, and cortisone (Fig. S1, Tables S3-S6). Four regimen-specific differences were also identified, with plasma urate and N-acetyl-1-methylhistidine significantly associated with PTB in untreated women only (Fig. 2B, Tables S3, S5). The overall increase in methionine sulfone levels in women who delivered preterm was largely driven by differences in the ZDV group (Fig. 2B). A sensitivity analysis conducted using newborn examination as the basis for preterm birth definition revealed distinct sets of significant features (Fig. S2A), although the regression estimates among features significant according to at least one of the measures were highly correlated (r = 0.76, p < 0.001, Fig. S2B). Regression models with elastic net regularization identified similar differences associated with preterm birth (Table S7).

### Markers of preterm birth in treatment-naïve women

**Fig. 3:**
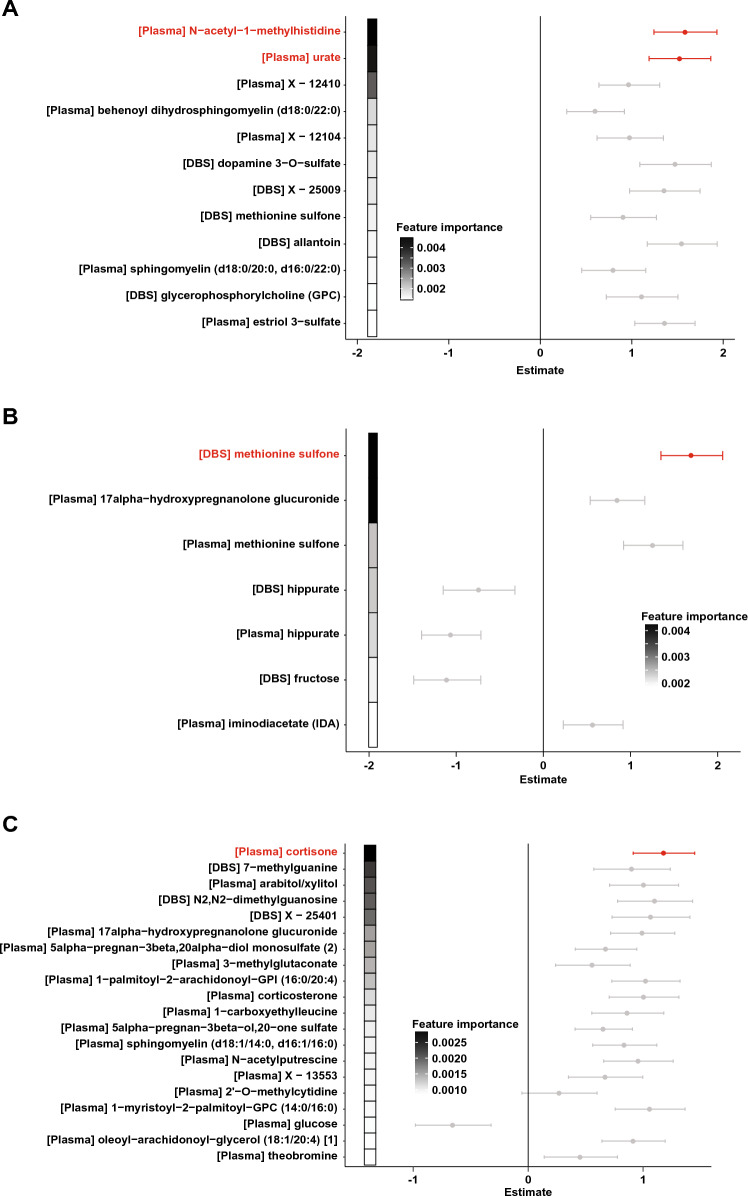
Maternal signatures of preterm birth. Random forests (RF) model for preterm birth in **a** untreated women, **b** women on zidovudine monotherapy, and **c** women on PI-ART. Features shown represent the sparse set selected by cross-validation and are ordered by decreasing importance in the RF model as indicated by shaded boxes on the left. Points and error bars show the coefficients and 95% confidence intervals from linear regression analysis of the same metabolite. Metabolites with positive estimates are increased in women who deliver preterm and metabolites with negative estimates are increased in women who deliver at term. Values in red were statistically significant in the linear regression analysis

To better characterize regimen-specific signatures of PTB, we next constructed random forests classification models separately for each treatment group. Multi-omics models utilizing both maternal plasma and DBS data achieved accuracies of 95.5%, 95.7%, and 80.7% in the untreated, ZDV monotherapy, and PI-ART groups, respectively (Fig. S3 and Table 2), with a mean accuracy of 89.5%. Plasma urate and N-acetyl-1-methylhistidine, identified by linear regression as markers of PTB, were also selected as predictive features in the random forests model for the untreated group (Fig. 3A).

**Table 2:** Random forests models

	Number of features	Accuracy	MCC^a^	AUC^b^	Sensitivity	Specificity
Multi-omics (n = 76)						
Untreated	12	95.45	0.9129	0.9669	1	0.9167
ZDV^c^	7	95.65	0.9161	0.9923	1	0.9091
PI-ART^d^	20	80.65	0.6193	0.9333	0.8571	0.7647

	Number of features	Accuracy	MCC	AUC	Sensitivity	Specificity
Plasma (n = 97)						
Untreated	5	92.86	0.8564	0.9641	0.9231	0.9333
ZDV	20	87.1	0.736	0.9872	0.85	0.9091
PI-ART	20	89.47	0.7922	0.9694	0.9375	0.8636

	Number of features	Accuracy	MCC	AUC	Sensitivity	Specificity
DBS (n = 76)						
Untreated	20	95.45	0.9129	0.9587	1	0.9167
ZDV	4	95.65	0.9161	0.9615	1	0.9091
PI-ART	20	83.87	0.6792	0.9208	0.8667	0.8125

Characteristics and performance of random forests models, multi-omic (maternal plasma + maternal DBS), maternal plasma, and maternal DBS for preterm birth in each treatment group

^a^Matthew’s correlation coefficient

^b^Area under the receiver operator characteristic curve

^c^Zidovudine monotherapy group

^d^Protease inhibitor-based antiretroviral therapy group

DBS levels of dopamine 3-O-sulfate, methionine sulfone, and allantoin were also selected as predictive markers of PTB (Fig. 3A). Separate models for PTB based on DBS- and plasma-derived metabolite profiles largely recapitulated these results (Figs. S4, S5 and Tables S9, S10). Levels of many of the identified features, including methionine sulfone, were positively correlated with creatinine levels (Fig. S6). Intriguingly, three uncharacterized compounds (‘X-12410’ and ‘X-12104’ in plasma, ‘X-25009’ in DBS) were also identified as markers of PTB.

### Metabolic predictors of preterm birth in women on zidovudine monotherapy

**Fig. 4:**
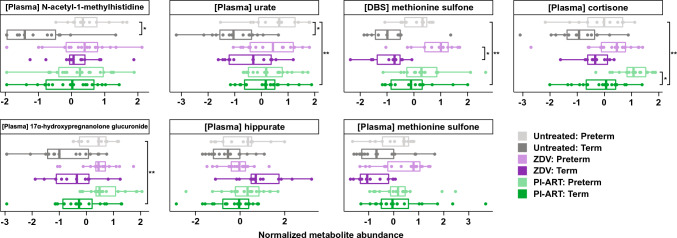
Selected metabolite abundances. Boxplots of normalized metabolite abundance values for selected features. Bold lines indicate medians, whiskers indicate 1.5*IQR (interquartile range) from first and third quartiles, and points indicate individual sample values. Statistically significant comparisons are marked with * FDR-adjusted p < 0.05, **FDR-adjusted p < 0.01 for comparison of PTB versus term delivery by treatment group (small brackets) or all PTB versus term delivery (large bracket)

In women on ZDV monotherapy, drastically altered levels of methionine sulfone and hippurate in both DBS and plasma, as well as 17α-hydroxypregnanolone glucuronide in plasma, were identified as key markers of PTB (Fig. 3B). Notably, 17α-hydroxypregnanolone glucuronide was significantly elevated in the preterm group as a whole (Figs. 4, S3) and appears to be a common plasma marker of PTB regardless of treatment. The overall elevation of methionine sulfone levels in women who delivered preterm appears to be driven primarily by large increases in the ZDV group and to a lesser extent in the PI-ART and untreated groups (Fig. 4). Hippurate levels were decreased in the ZDV group but not the PI-ART and untreated groups (Figs. 3, 4).

### Preterm birth in women on protease inhibitor-based ART

The classification model for PTB in women on PI-based ART did not perform as well as those in the other groups (Fig. 2). However, a large number of steroid compounds including cortisone, 17alpha-hydroxypregnanolone glucuronide, 5alpha-pregnan-3beta, 20alpha-diol monosulfate, and 5alpha-pregnan-3beta-ol,20-one sulfate were selected as predictive features (Fig. 3C). DBS levels of two compounds involved in purine metabolism, 7-methylguanine and N2,N2-dimethylguanosine, were also strongly associated with PTB. The increase in cortisone levels among women who delivered preterm is striking (Fig. 4) and may reflect higher stress levels in women who deliver preterm.

### Profiles of preterm birth in the infant

In addition to identifying potentially predictive markers of PTB in maternal substrates, we also wanted to investigate the impact of PTB on the newborn infant. An initial analysis revealed strong segregation between metabolite profiles collected from infants either within the first 3 days of life or at a later time up to 1 month of age (Fig. S7), likely due to contributions from infant metabolism. Therefore, we focused our analysis on a subset of n = 56 infants who were sampled within the first 3 days of life (Table 3; Fig. S1).

**Table 3:** Demographic characteristics related to infant analyses

	ZDV—term	ZDV—preterm	PI-ART—term	PI-ART—preterm	p
n	7	8	21	20	
Sex = Male (%)	2 (28.6)	2 (25.0)	12 (57.1)	8 (40.0)	0.328
Birthweight (mean (SD), g)	2895 (275)	2576 (482)	2798 (455)	2477 (478)	0.07
Weight at 1 week (mean (SD), g)	3328 (314)	2958 (611)	3112 (528)	2739 (596)	0.078
Gestational age at start of therapy (mean (SD), weeks)	29.4 (3.7)	23.7 (5.0)	25.7 (5.0)	25.9 (4.7)	0.155
Country (%)					0.095
India	0 (0.0)	0 (0.0)	2 (9.5)	2 (10.0)	
Malawi	5 (71.4)	5 (62.5)	1 (4.8)	5 (25.0)	
South Africa	1 (14.3)	3 (37.5)	14 (66.7)	9 (45.0)	
Uganda	1 (14.3)	0 (0.0)	3 (14.3)	3 (15.0)	
Zambia	0 (0.0)	0 (0.0)	1 (4.8)	1 (5.0)	
Gestational age at delivery (mean (SD), weeks)	41.9 (5.7)	31.2 (4.1)	39.8 (1.9)	33.3 (2.0)	** < 0.001**
Infant age in days (mean (SD))	1.1 (0.7)	1.6 (0.9)	1.1 (0.7)	1.8 (1.0)	**0.044**

Bold values indicate p < 0.05

Demographics of the mother-infant dyads used for analysis of infant samples. p values are derived from χ^2^ and one-way ANOVA tests for categorical and continuous variables, respectively

**Fig. 5:**
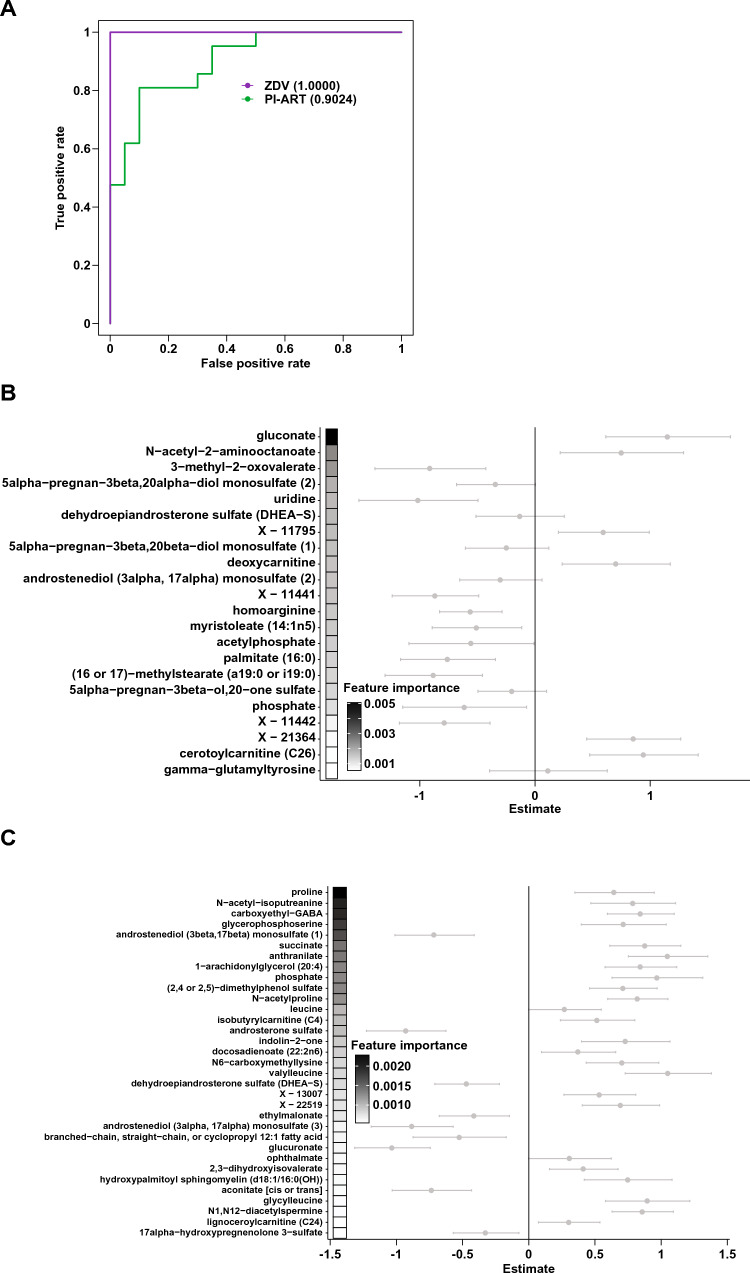
Infant signatures of preterm birth. Random forests (RF) model for preterm birth using infant DBS-derived metabolite profiles. **a** Receiver-operator characteristic curves for classification models of birth status for each treatment regimen as indicated. Numbers in parentheses indicate the area under the ROC curve (AUC). **b**–**c** RF models for preterm birth in infants exposed to (**b**) zidovudine monotherapy and **c** PI-ART in utero. Features are ordered by decreasing importance in the RF model as shown by shaded boxes on the left. Points and error bars show the coefficients and 95% confidence intervals from linear regression analysis of the same metabolite. Metabolites with positive estimates are increased in preterm infants and metabolites with negative estimates are increased in term infants

As with the maternal profiles, preterm versus term birth, antiretroviral regimen, and country of origin were identified as significant drivers of overall variation in the infant profiles (Table S2). Even though this analysis was performed only on infants within the first 3 days of life, the infant’s age in days still explained 3.7% of the overall variation, suggesting that the infant metabolome changes rapidly after delivery. Linear regression did not identify any significantly altered compounds in either the ZDV monotherapy or PI-ART treatment arms, nor among all preterm infants as a whole (Tables S11, S12). Although classification models for preterm birth are not particularly insightful in this context, we still utilized this approach as an alternative means of feature selection (Fig. 5 and Table S13, 90.0% and 89.3% accuracy for the ZDV and PI-ART groups, respectively). Notably, gluconate as well as a plethora of progestin and androgenic steroid compounds including dehydroepindrosterone sulfate (DHEA-S) were selected as features in the ZDV model (Fig. 5B). In the PI-ART model, the top discriminatory features included proline, N-acetyl-isoputreanine, and carboxylethyl-GABA (Fig. 5C), all of which are involved in amino acid metabolism.

## Discussion

We applied an untargeted metabolomics approach to characterize metabolic signatures of preterm birth in a cohort of 100 WLH prior to treatment and on two different treatment regimens. Linear regression and random forests classification models revealed both shared and regimen-specific markers of PTB.

Plasma N-acetyl-1-methylhistidine and urate were significantly increased in untreated women who delivered preterm and to a lesser extent in the preterm groups of the two other groups. N-acetyl-1-methylhistidine is a known marker of chronic kidney disease, and reduced kidney function has been associated with pregnancy complications including PTB (Kendrick et al., 2015; Yu et al., 2014). Maternal urate levels have also been associated with adverse perinatal outcomes including PTB in hypertensive pregnancy (Hawkins et al., 2012; Roberts et al., 2005).

Methionine sulfone and hippurate were found to be significantly altered in women on ZDV monotherapy who delivered preterm. Two recent metabolomic studies found higher levels of methionine sulfone in people living with HIV versus healthy controls (Babu et al., 2019) and specifically as an effect of long-term ART (Peltenburg et al., 2018). At the time of sampling, our cohort had exposure to only a short duration of treatment and therefore it is possible that the oxidative stress indicated by this metabolite had only started to accumulate. Methionine sulfone was also found to be correlated with creatinine levels as measured by the metabolomics platform. Creatinine levels were previously found in a separate study to be associated with increased risk of provider-initiated but not spontaneous preterm births (Harel et al., 2020). Altogether, the magnitude of the increase observed in the ZDV group as well as the association with creatinine levels suggests that methionine sulfone may function pleiotropically with respect to PTB. Hippurate is a product of shared metabolism by host and microbial pathways and has been associated with consumption of fruits, vegetables, and whole grains (Lees et al., 2013; Pallister et al., 2017). Recent studies have identified hippurate as a key biomarker of gut microbial diversity, the loss of which is implicated in a number of diseases including metabolic syndrome, inflammatory bowel disease, and Crohn’s disease (Pallister et al., 2017; Williams et al., 2009, 2010). It would be interesting to speculate that the decreased levels of hippurate seen in women who deliver preterm reflect a paradigm of gut microbiota dysbiosis, but additional investigations are necessary given the lack of dietary data and small sample size of the present study.

Drastic remodeling of progesterone and other steroid pathways was observed in women who delivered preterm. A recent metabolomic study of preterm birth among women not living with HIV also observed significant differences in lipid and steroid-related metabolites (Manuck et al., 2021), although there was little overlap between the specific features identified. This may be due to differences in the timing of sample collection (median of 19 weeks’ gestation versus 31 weeks in our study) as well as patient demographics (Western versus primarily African). In our study, 17α-hydroxypregnanolone glucuronide was increased in all treatment groups, suggesting that is it a key marker of PTB in general. Relatively little is known about 17α-hydroxypregnanolone glucuronide as opposed to the pregnenolone form that is an intermediate in sex steroid biosynthesis pathways, but a speculative pathway for its production suggests that it reflects progesterone metabolism as opposed to availability. Levels of two other pregnanolone isomers, 5alpha-pregnan-3beta, 20alpha-diol monosulfate, and 5alpha-pregnan-3beta-ol,20-one sulfate, were decreased in women on PI-based ART compared to those on ZDV or sampled prior to treatment. However, these two isomers were found at higher levels in women who delivered preterm versus term taking PI-ART. These two compounds are reduced progesterone metabolites and thus may reflect protease inhibitor-mediated decreases in progesterone levels (Papp et al., 2015, 2016). Progesterone supplementation by multiple forms including 17α-hydroxyprogesterone caproate (17-OHPC) has been widely used prophylactically to reduce the risk of PTB in at-risk women (Committee on Practice Bulletins-Obstetrics TACoO, Gynecologists, 2012; Meis et al., 2003). However, recent clinical trials have failed to identify any effect of 17-OHPC supplementation on rates of PTB (Blackwell et al., 2020; Coler et al., 2021; Nelson et al., 2017; Price et al., 2019) and the US Food and Drug Administration (FDA) has recently withdrawn its approval of hydroxyprogesterone caproate injection to reduce the risk of preterm birth. Notably, these larger studies were conducted in widely disparate populations from both developed and developing nations, including a cohort of HIV-infected Zambian women (Price et al., 2019, 2021).

Although we observed a consistent elevation of 17α-hydroxypregnanolone glucuronide levels among women who delivered preterm across all three groups, the overlapping nature of the abundance values suggests significant underlying variation in progesterone metabolism. One possibility is that the efficacy of 17-OHPC supplementation on sPTB may be limited to a subset of women who have lower baseline levels of progesterone availability.

Decreased hippurate levels were observed specifically among women who delivered preterm in the ZDV group, with the trend actually reversing among the untreated and PI-ART groups. Although investigations into the role of commensal microbial communities on PTB have largely focused on the urogenital tract (Staude et al., 2018), there is some evidence that gut microbial dysbiosis is also involved, either as a source for translocation or more generally as a driver of systemic inflammation (Dahl et al., 2017; Staude et al., 2018). Given the association between hippurate and gut microbial diversity (Pallister et al., 2017), it would certainly be informative to assess the gut microbiota in the context of preterm birth and the various treatment regimens.

Use of infant DBS samples for metabolomics in the first 3 days of life identified infants that were preterm with approximately 90% accuracy in the group exposed to ZDV and the group exposed to PI-ART in this small sample set. This suggests that metabolomics on infant DBS shortly after birth has potential for development as a method for post-natal gestational dating.

### Strengths and limitations

The strengths of this study are the utilization of an untargeted metabolomics platform, which allows for unbiased interrogation of hundreds of biochemical compounds. This powerful approach does however require appropriate statistical treatment to limit the appearance of false positive results as well as a means to identify the most salient markers for the outcome of interest. The random forests method inherently encodes feature selection via cross-validation, thereby allowing for the identification of a sparse set of markers for the outcome of interest. Furthermore, a consensus approach using two different analytic methods (linear regression and random forests) with different underlying assumptions gives additional confidence to the markers identified by both. Finally, by focusing on the most robust set of features, we can more easily infer biological plausibility and translation to intervention as opposed to a larger panel of hundreds of markers.

A major limitation of this pilot study is the lack of data regarding the circumstances of PTB; notably, it is possible that some of the preterm births in this study were iatrogenic as opposed to spontaneous. Such cases, as well as other confounders such as diet and socioeconomic status, could introduce additional variation and limit the power of an already small sample size to detect robust markers of sPTB. Additionally, this sample size is also based on even matching between cases and controls, which does not reflect the true population distribution and therefore could lead to excessive sensitivity to preterm deliveries. Furthermore, the small sample size and large number of classifiers built in this study are susceptible to overfitting. It will be vitally important to test these preliminary findings in an independent validation cohort, and such efforts are underway. The dynamic, complex, and dramatic physiological changes associated with pregnancy also complicate our ability to detect robust signals from a cross-sectional study design. Rather, it may be necessary to characterize signatures of preterm birth as differences in metabolic trajectories over the course of pregnancy. Such a design would also obviate the need to identify the correct timing window during gestation at which the signature of preterm birth is most robust while also weighing the potential for earlier intervention. All participants in this study were on ZDV-based regimens, except a single PI-ART participant; therefore, the changes identified in the PI-ART group may reflect exposure to any component of the regimen. However, given the increased incidence of PTB with protease inhibitors as well as the known effects on progesterone metabolism, we believe these reflect true metabolic alterations in pregnant women on PI-based ART. Finally, the samples used in this study were collected during a period when ZDV monotherapy and PI-ART was still in use. While protease-inhibitor based regimens have been replaced by newer antiretroviral regimens, the association of PI-based ART with an increased rate of preterm birth allows the use of the antiretroviral agent as a probe to better understand unique and/or additional metabolic derangements associated with PTB. Understanding the potential interaction between these regimens, newer antiretroviral regimens, and PTB will be important for translation to clinical practice.

## Conclusions

Caveats aside, our preliminary study provides a number of valuable insights into potential mechanisms underlying spontaneous PTB in a complex, high-risk population. Several of the metabolites identified as markers of PTB here have been previously implicated in other cohorts but studies to better understand the mechanisms and pathways behind their modulation are needed. Potential links to the gut microbiota vis-à-vis hippurate co-metabolism and to protease inhibitor-associated disruption of progesterone availability also warrant further investigation. Finally, our findings provide a proof of concept for the use of untargeted metabolomics as a tool for hypothesis generation and biomarker discovery in complex disease processes.

### Supplementary Information

Below is the link to the electronic supplementary material.

Supplementary file1 (DOCX 954 KB)

Supplementary file2 (DOCX 1469 KB)

Supplementary file3
Supplementary Table 1. Demographics of the mother-infant dyads used for analysis of maternal DBS samples. P-values are derived from χ2 and one-way ANOVA tests for categorical and continuous variables, respectively.
Supplementary Table 2. PERMANOVA results for maternal plasma, maternal DBS, and infant DBS metabolite profiles.
Supplementary Table 3. Linear regression analysis for maternal plasma metabolites stratified by treatment group.
Supplementary Table 4. Linear regression analysis for maternal plasma metabolites averaged across treatment groups.
Supplementary Table 5. Linear regression analysis for maternal DBS metabolites stratified by treatment group.
Supplementary Table 6. Linear regression analysis for maternal DBS metabolites averaged across treatment groups.
Supplementary Table 7. Elastic net regression analysis for maternal plasma and DBS profiles.
Supplementary Table 8. Features selected in random forests models of preterm birth using data from both maternal plasma and DBS profiles.
Supplementary Table 9. Features selected in random forests models of preterm birth from maternal plasma profiles.
Supplementary Table 10. Features selected in random forests models of preterm birth from maternal DBS profiles.
Supplementary Table 11. Linear regression analysis for infant DBS metabolites stratified by treatment group.
Supplementary Table 12. Linear regression analysis for infant DBS metabolites averaged across treatment groups.
Supplementary Table 13. Features selected in random forests models of preterm birth from infant DBS profiles. (XLSX 1298 KB)

Supplementary file4 (DOCX 76 KB)

## Data Availability

Data and analysis code are available to all interested researchers upon reasonable request to the IMPAACT Statistical and Data Management Center’s data access committee (email address: sdac.data@fstrf.org) with the agreement of the IMPAACT Network.
