# Supplementary material for: Metabolomic profiling of preterm birth in pregnant women living with HIV
Source: Metabolomics. 2023 Oct 25;19(11):91. doi: 10.1007/s11306-023-02055-1 (PMC10600291; doi:10.1007/s11306-023-02055-1)

**Online Resource 1. Supplemental Results describing the exclusion of 21 DBS samples due to storage-related batch effects.**

**Supplemental Results**

*Batch effect in maternal dried blood spot (DBS) samples from a single study site*

Untargeted metabolomics was performed on maternal plasma, maternal dried blood spots (DBS), and infant DBS from 100 mother-infant dyads. Standard dimensionality reduction and visualization techniques (e.g. principal components analysis, t-SNE) were used for initial sample QC and outlier detection. A subset of DBS samples from Malawi were observed to segregate from all others with sufficient signal to drive the second principal component (Figure SR1) and an even stronger effect was observed on t-SNE (Figure SR2). Notably, this effect was not observed in maternal plasma profiles (Figures SR3 and SR4) and all of the affected samples had accessions numbering 6****** (6-series) as opposed to 3****** (3-series). This prompted us to inquire with the study coordinators, who identified that all of the samples numbered from the 6-series in Malawi were indeed collected and stored at a single site. Further investigation suggested that these DBS samples may have been exposed to higher levels of humidity, and levels of compounds identified by Metabolon as particularly susceptible to stability effects were indeed altered in these samples (Figure SR5). Altogether, we felt that this provided compelling and sufficient evidence to justify exclusion of the DBS samples of these 21 mother-infant dyads from all further analysis.


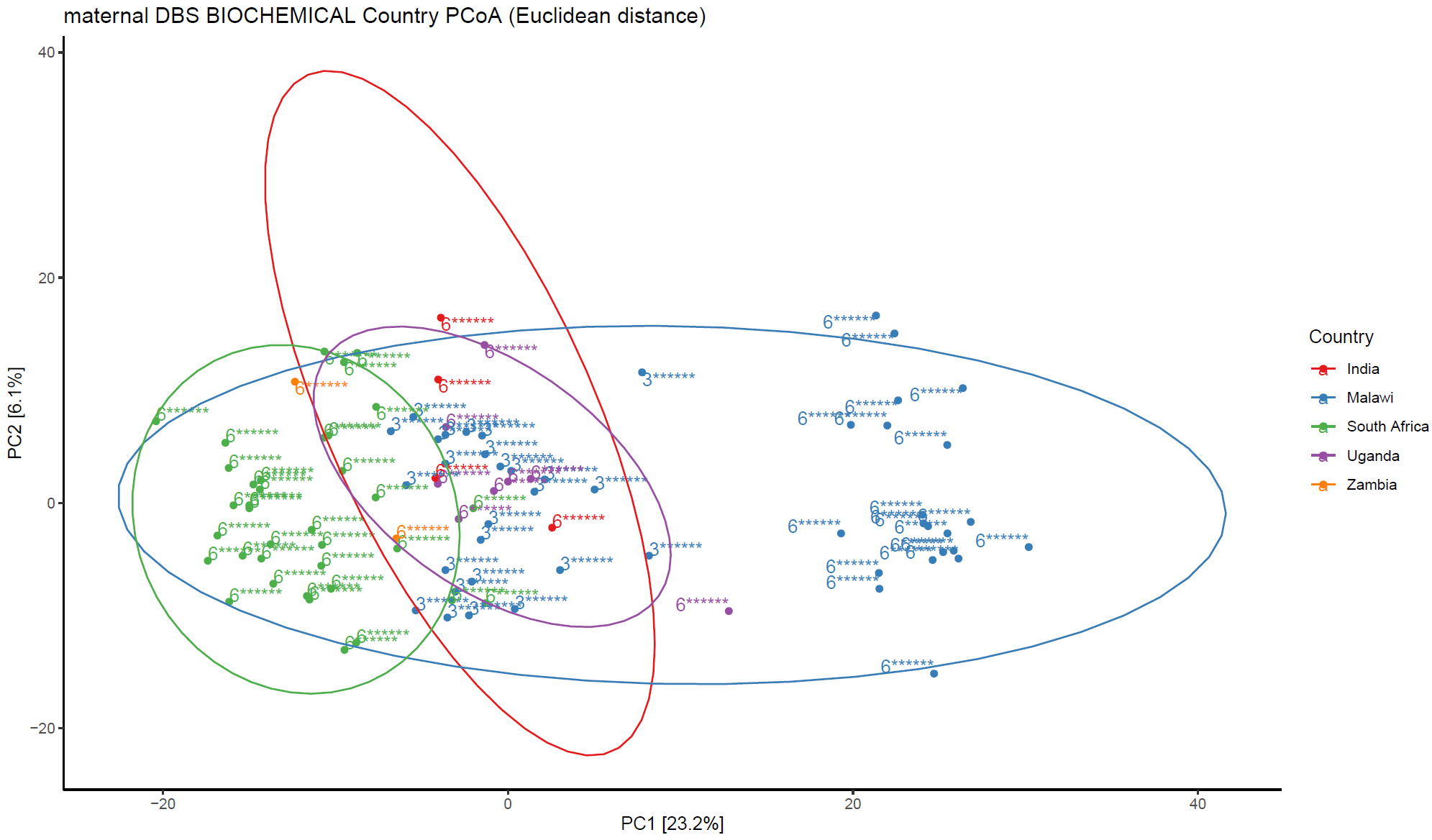
Figure SR1. Principal components analysis (PCA) plot of maternal DBS samples colored by country. Numbers in brackets along axes indicate percentage of overall variation explained.

Figure SR2. t-SNE projection of maternal DBS samples colored by country


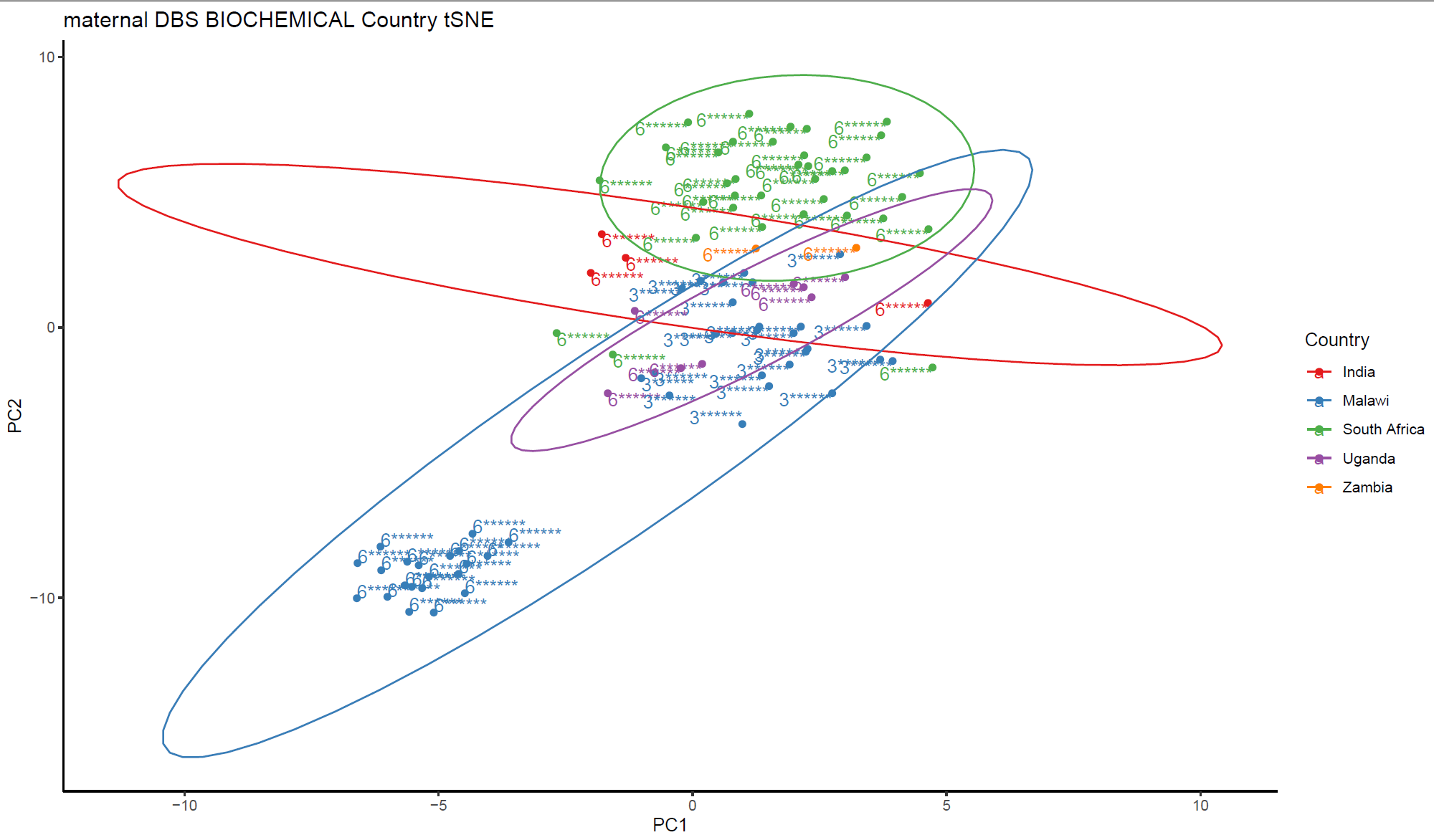


Figure SR3. Principal components analysis (PCA) plot of maternal plasma samples colored by country. Numbers in brackets along axes indicate percentage of overall variation explained.


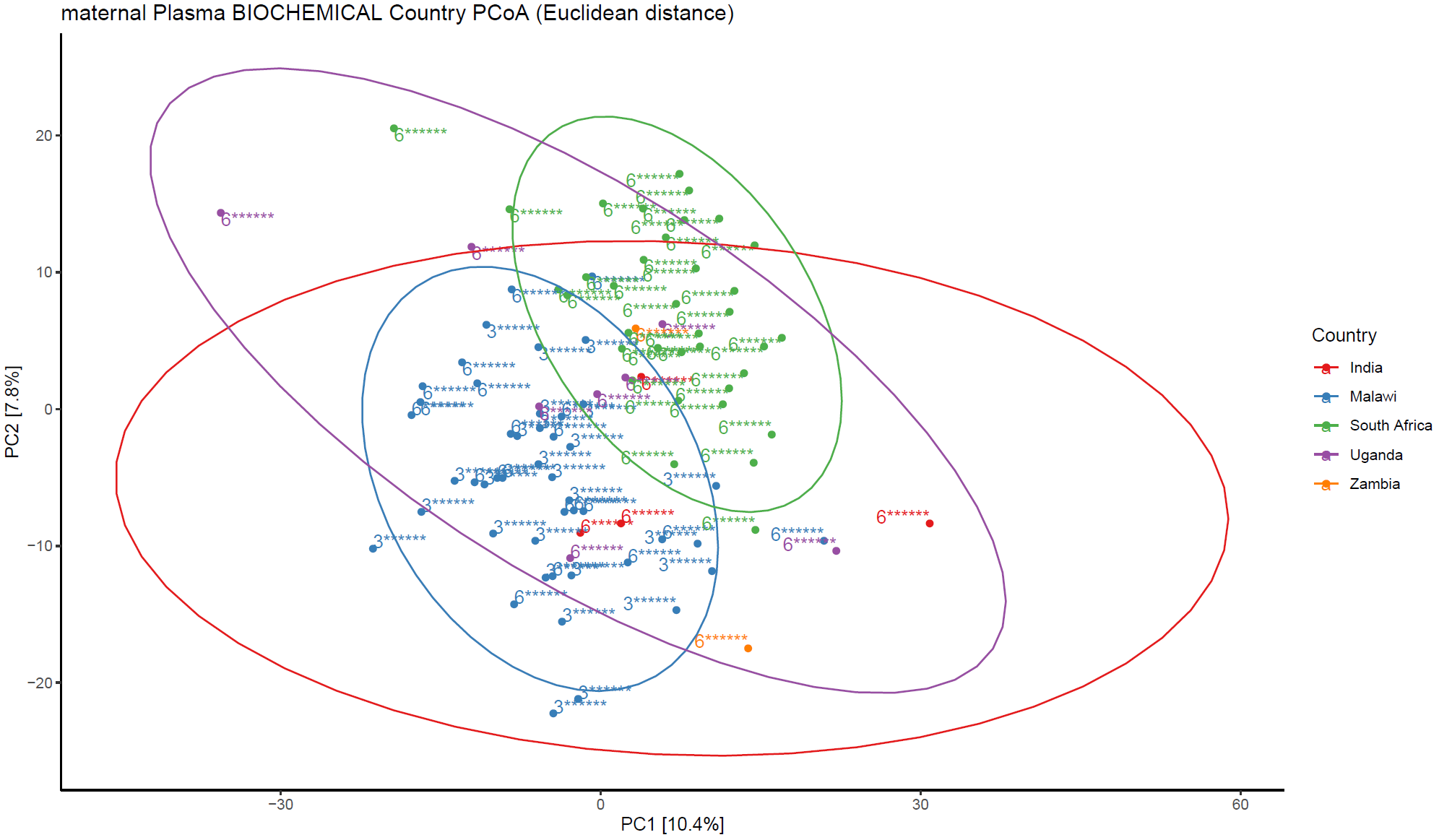


Figure SR4. t-SNE projection of maternal plasma samples colored by country.


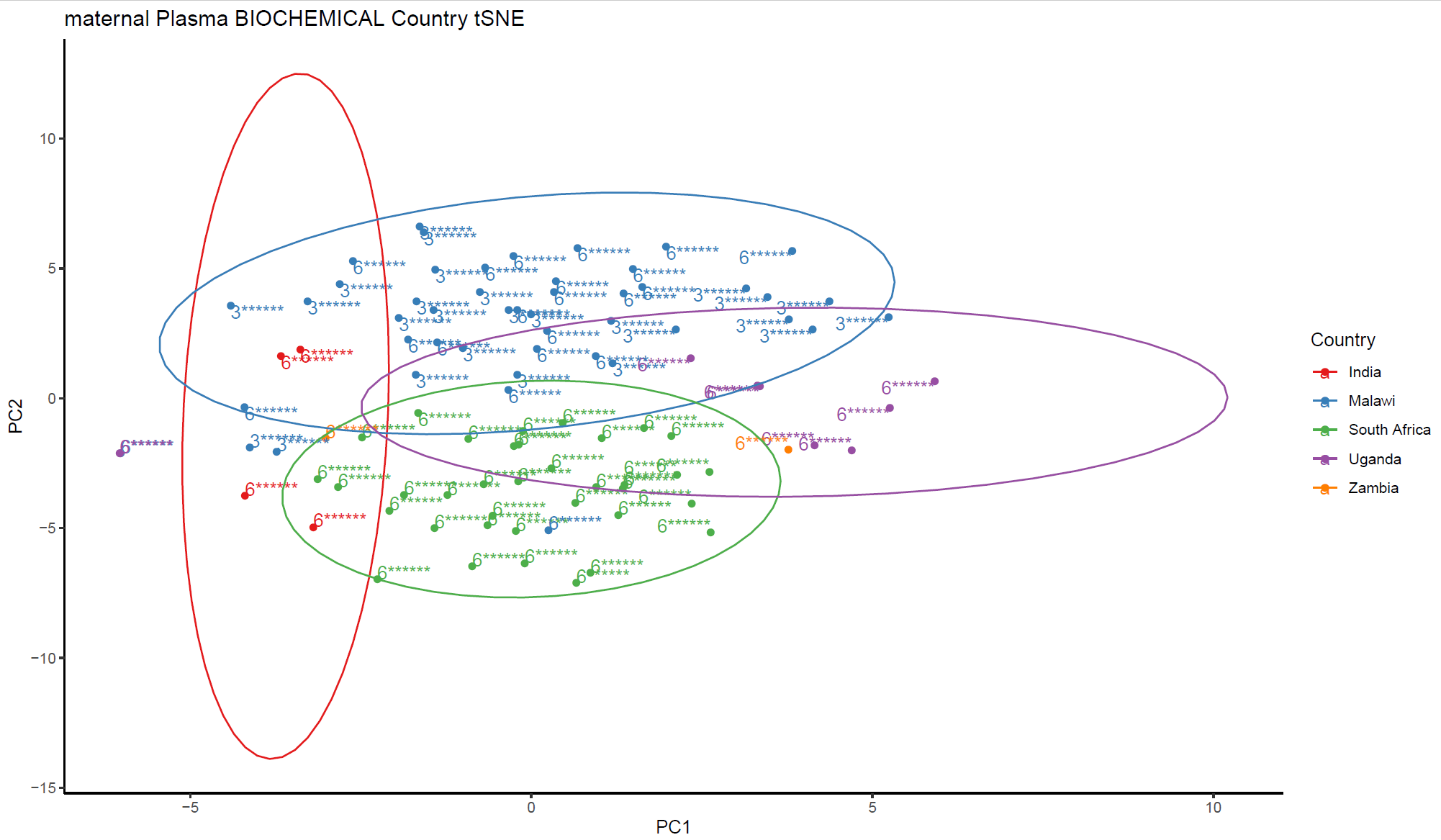


Figure SR5. Distribution of DBS normalized abundance values for metabolites selected in random forests model classifying between study sites in Malawi, with available stability data. Note that thioproline was observed to be degraded at room temperature whereas all others were concentrated.


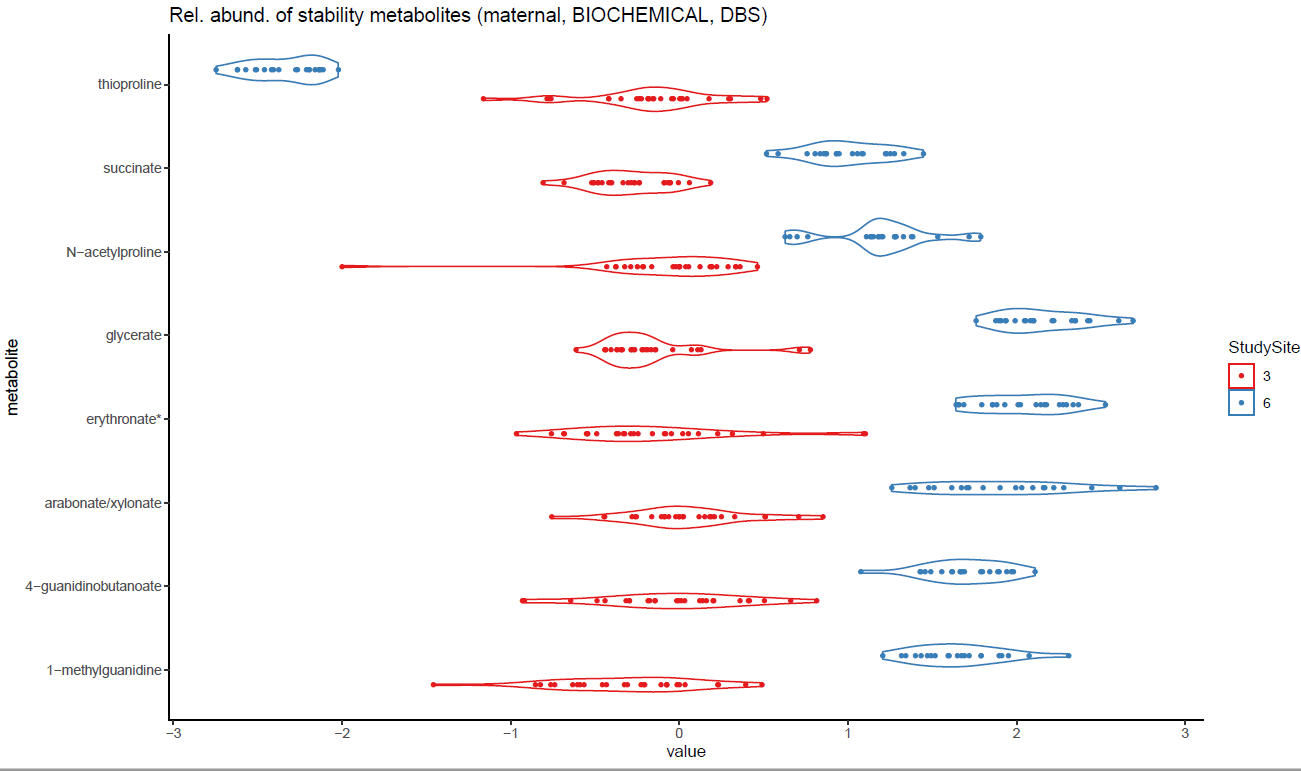

Supplement: Supplementary file 1 — Supplementary file1 (DOCX 954 KB) [file 11306_2023_2055_MOESM1_ESM.docx]
