# Supplementary material for: Metabolomic profiling of preterm birth in pregnant women living with HIV
Source: Metabolomics. 2023 Oct 25;19(11):91. doi: 10.1007/s11306-023-02055-1 (PMC10600291; doi:10.1007/s11306-023-02055-1)
Supplement: Supplementary file 2 — Supplementary file2 (DOCX 1469 KB) [file 11306_2023_2055_MOESM2_ESM.docx]

**Online Resource 2 – Supplemental Figures**

**Metabolomic profiling of preterm birth in pregnant women living with HIV**

Nicole H. TOBIN, MD^1^

Aisling MURPHY, MD^2^

Fan LI, PhD^1^

Sean S. BRUMMEL, PhD^3^

Mary Glenn FOWLER, MD MPH^4^

James A. MCINTYRE, FRCOG^5,6^

Judith S CURRIER, MD^7^

Tsungai CHIPATO, FRCOG^8^

Patricia M. FLYNN, MD^9^

Luis A. GADAMA^10^

Friday SAIDI, MD^11^

Clemensia NAKABIITO^12^

Brian J. KOOS, MD DPhil^2^

Grace M. ALDROVANDI, MD CM^1,*^

And the IMPAACT 1077BF/1077FF PROMISE Study Team

^1^Division of Infectious Diseases, Department of Pediatrics, David Geffen School of Medicine at the University of California, Los Angeles, California

^2^Department of Obstetrics and Gynecology, David Geffen School of Medicine at the University of California, Los Angeles, California

^3^Center for Biostatistics in AIDS Research, Harvard T.H. Chan School of Public Health, Boston, Massachusetts

^4^ Department of Pathology, Johns Hopkins U. School of Medicine, Baltimore, Maryland

^5^Anova Health Institute, Johannesburg, South Africa

^6^School of Public Health and Family Medicine, University of Cape Town, Cape Town, South Africa

^7^Division of Infectious Diseases, Department of Internal Medicine, David Geffen School of Medicine at the University of California, Los Angeles, California

^8^University of Zimbabwe College of Health Sciences

^9^Department of Infectious Diseases, St. Jude Children’s Research Hospital, Memphis, Tennessee

^10^ Department of Obstetrics and Gynecology, Johns Hopkins Research Project, Kamuzu University of Health Sciences, Malawi

^11^University of North Carolina Project Malawi, Lilongwe, Malawi
^12^MU-JHU Research Collaboration (MUJHU CARE LTD) CRS, Kampala, Uganda

*Correspondence to [galdrovandi@mednet.ucla.edu](mailto:galdrovandi@mednet.ucla.edu)


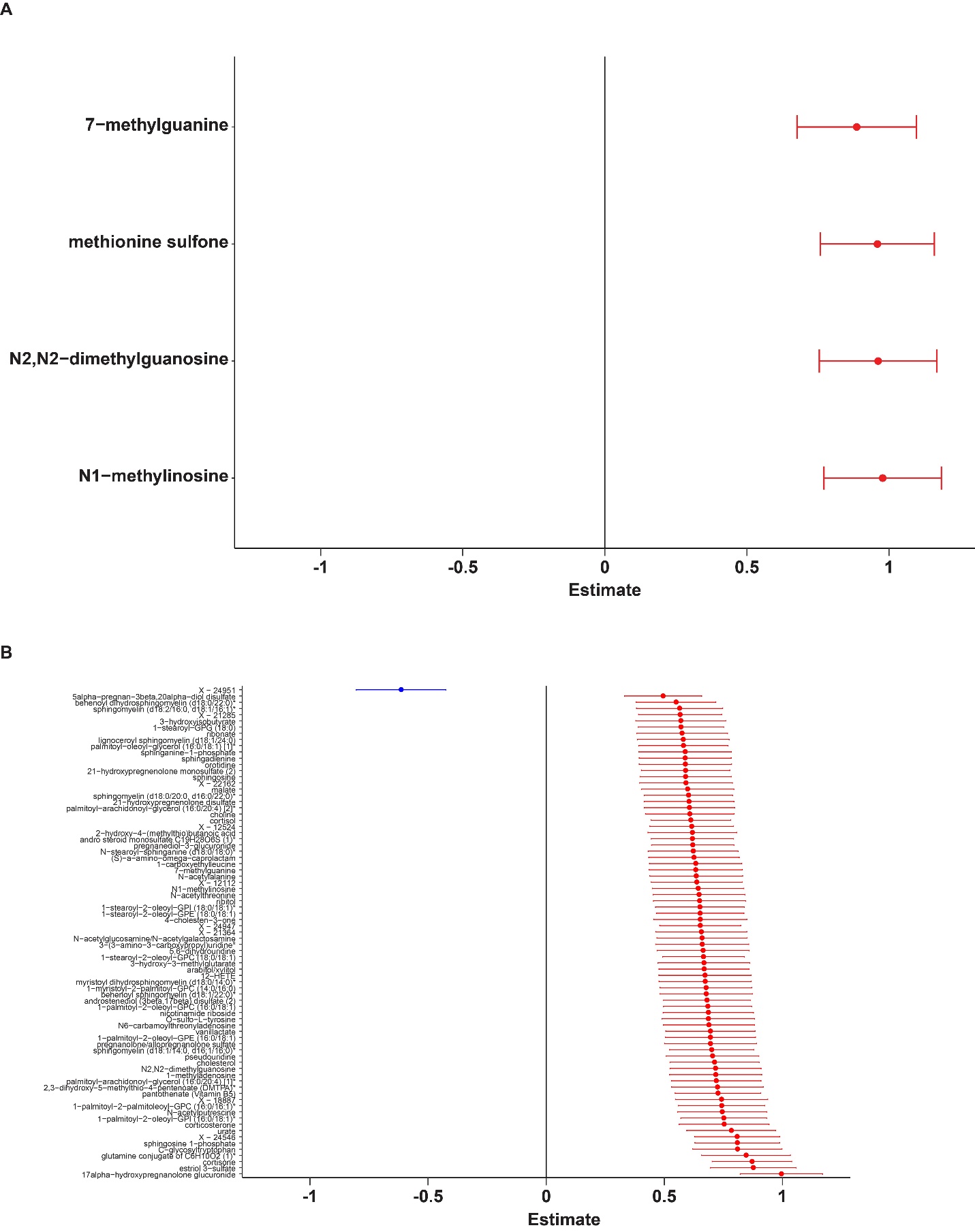
Figure S1. Coefficients from linear regression analysis of (a) maternal DBS and (b) plasma metabolites averaged across all treatment regimens. Metabolites with positive estimates are increased in women who deliver preterm and metabolites with negative estimates are increased in women who deliver at term. Only statistically significant coefficients are shown.


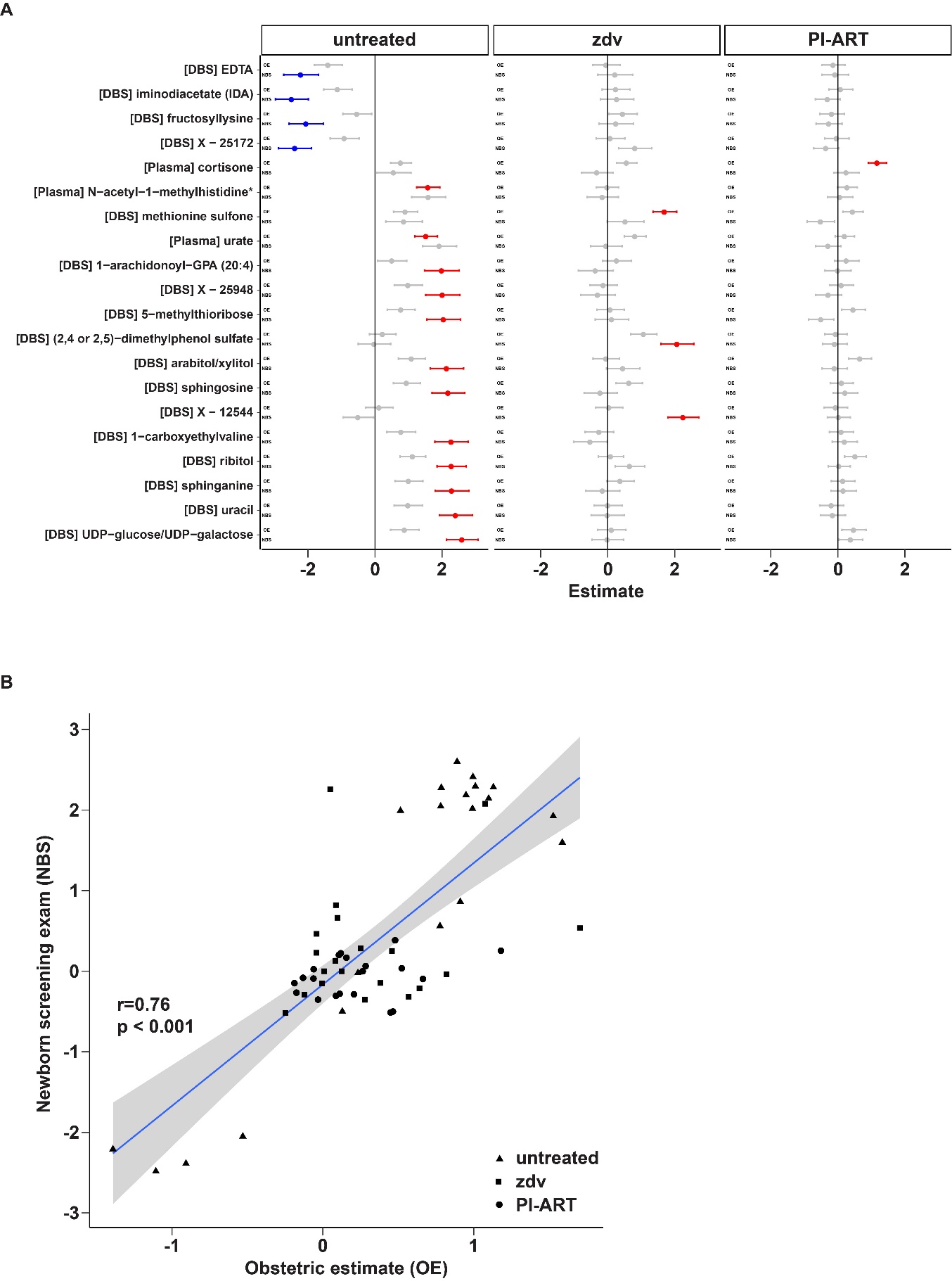


Figure S2. Sensitivity analysis with pediatric estimates of preterm birth via newborn examination. (a) Coefficients from linear regression analysis of maternal metabolites stratified by treatment regimen with the preterm birth outcome defined by either obstetric estimate (OE) or newborn screening exam (NBS). (b) Correlation plot of the coefficients shown in (a).


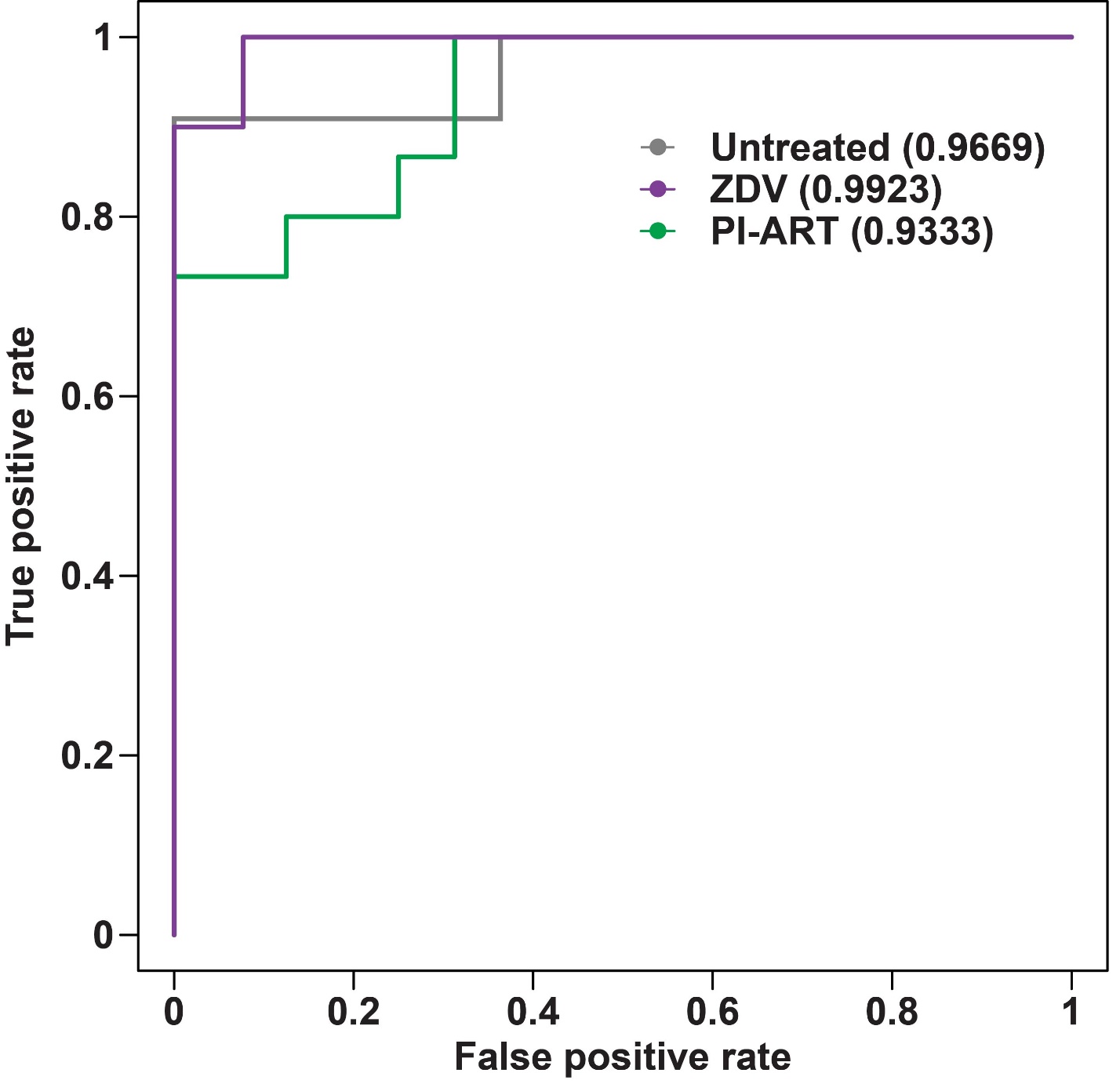


Figure S3. Receiver-operator characteristic curves (ROC) for random forests classification models of birth status for each treatment regimen as indicated. Numbers in parentheses indicate the area under the ROC curve (AUC).


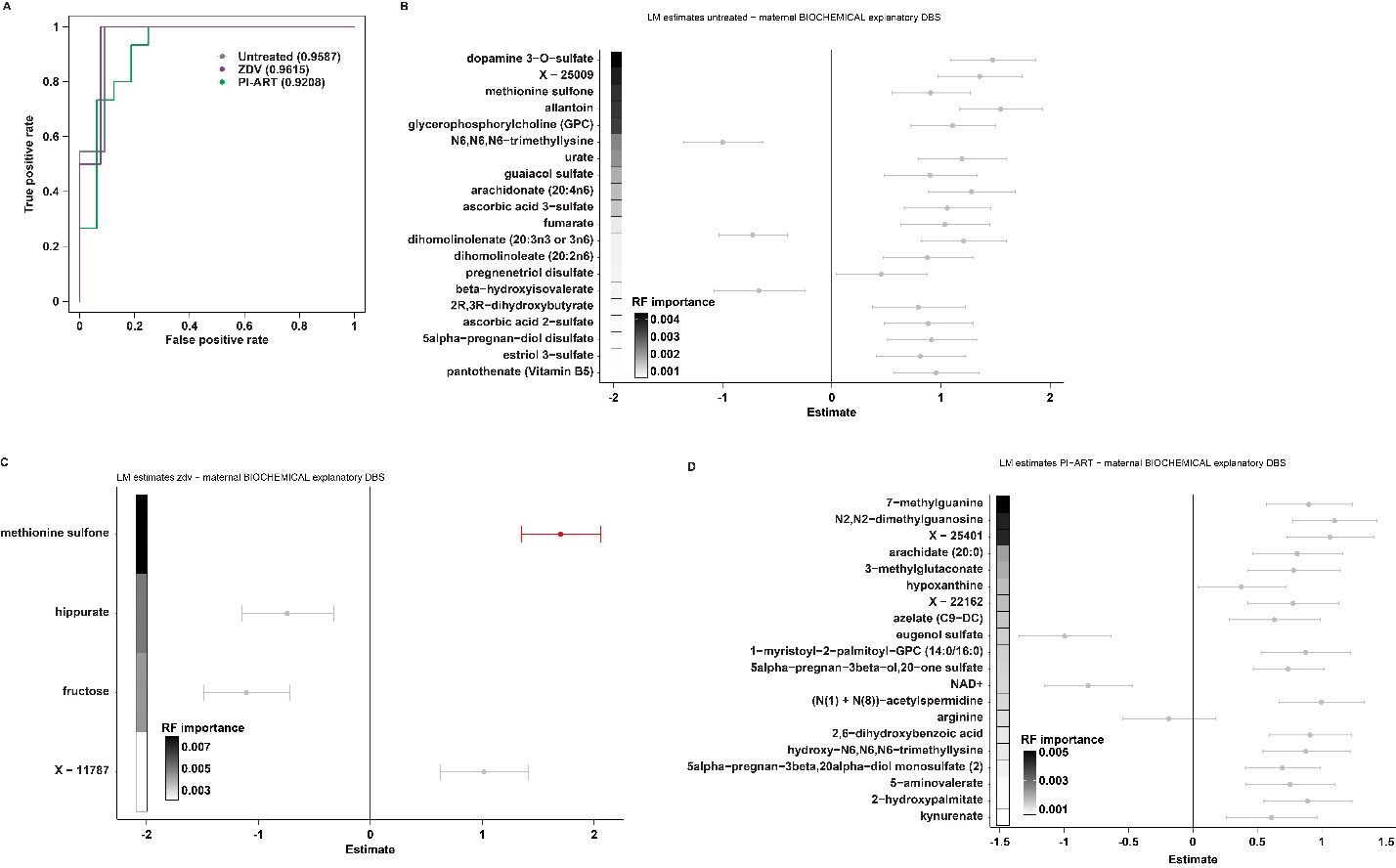


Figure S4. Random forests classification model of birth status using maternal DBS-derived metabolite profiles. (a) Receiver-operator characteristic curves for each treatment regimen as indicated. Numbers in parentheses indicate the area under the ROC curve (AUC). (b-d) Features shown represent the sparse set selected by cross-validation in the (b) untreated, (c) zidovudine monotherapy, and (d) PI-ART groups, and are ordered by decreasing importance in the RF models as indicated by shaded boxes on the left. Points and error bars show the coefficients and 95% confidence intervals from linear regression analysis of the same metabolite. Metabolites with positive estimates are increased in women who deliver preterm and metabolites with negative estimates are increased in women who deliver at term. Values in red were statistically significant in both analyses.


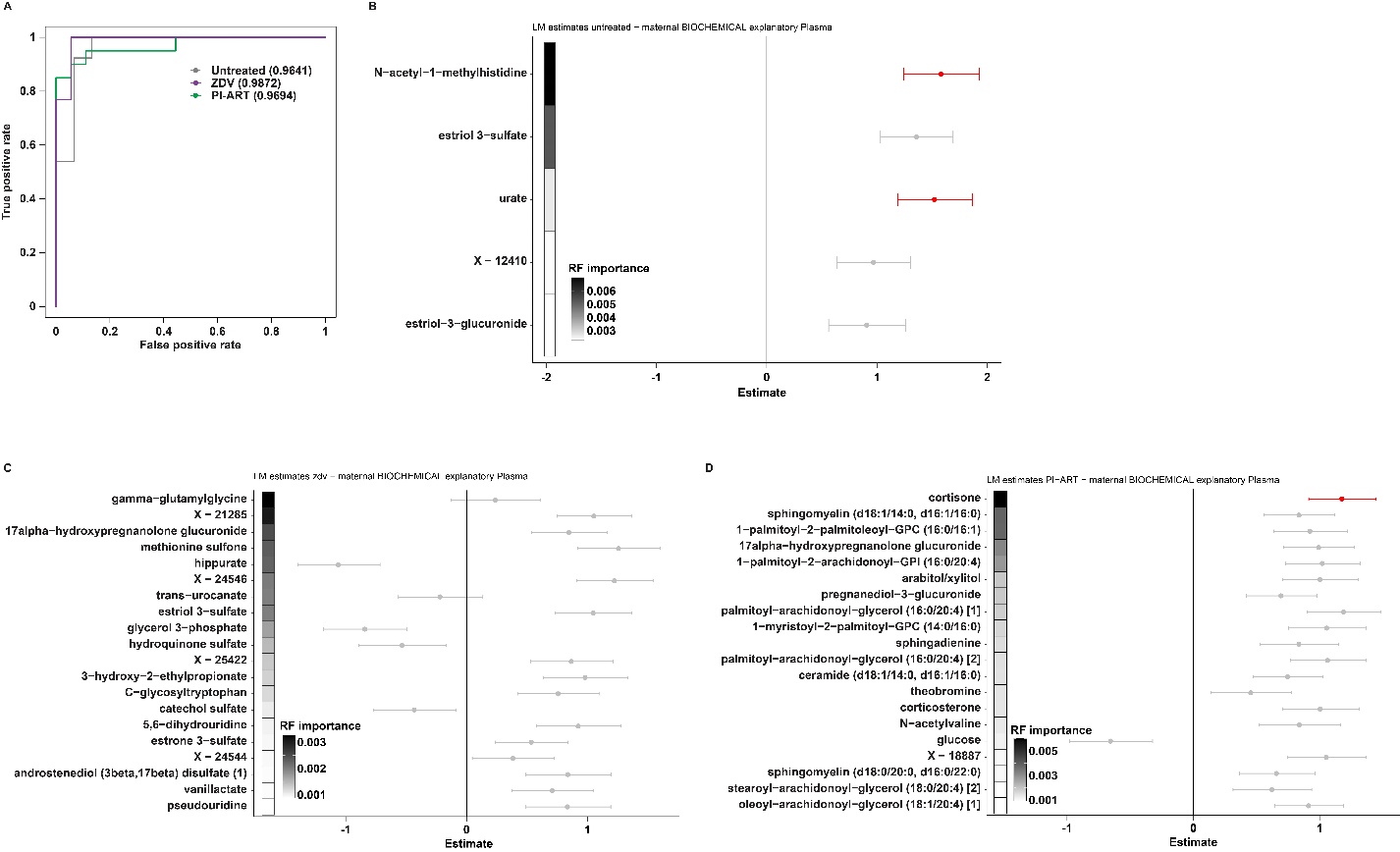


Figure S5. Random forests classification model of birth status using maternal plasma-derived metabolite profiles. (a) Receiver-operator characteristic curves for each treatment regimen as indicated. Numbers in parentheses indicate the area under the ROC curve (AUC). (b-d) Features shown represent the sparse set selected by cross-validation in the (b) untreated, (c) zidovudine monotherapy, and (d) PI-ART groups, and are ordered by decreasing importance in the RF models as indicated by shaded boxes on the left. Points and error bars show the coefficients and 95% confidence intervals from linear regression analysis of the same metabolite. Metabolites with positive estimates are increased in women who deliver preterm and metabolites with negative estimates are increased in women who deliver at term. Values in red were statistically significant in both analyses.


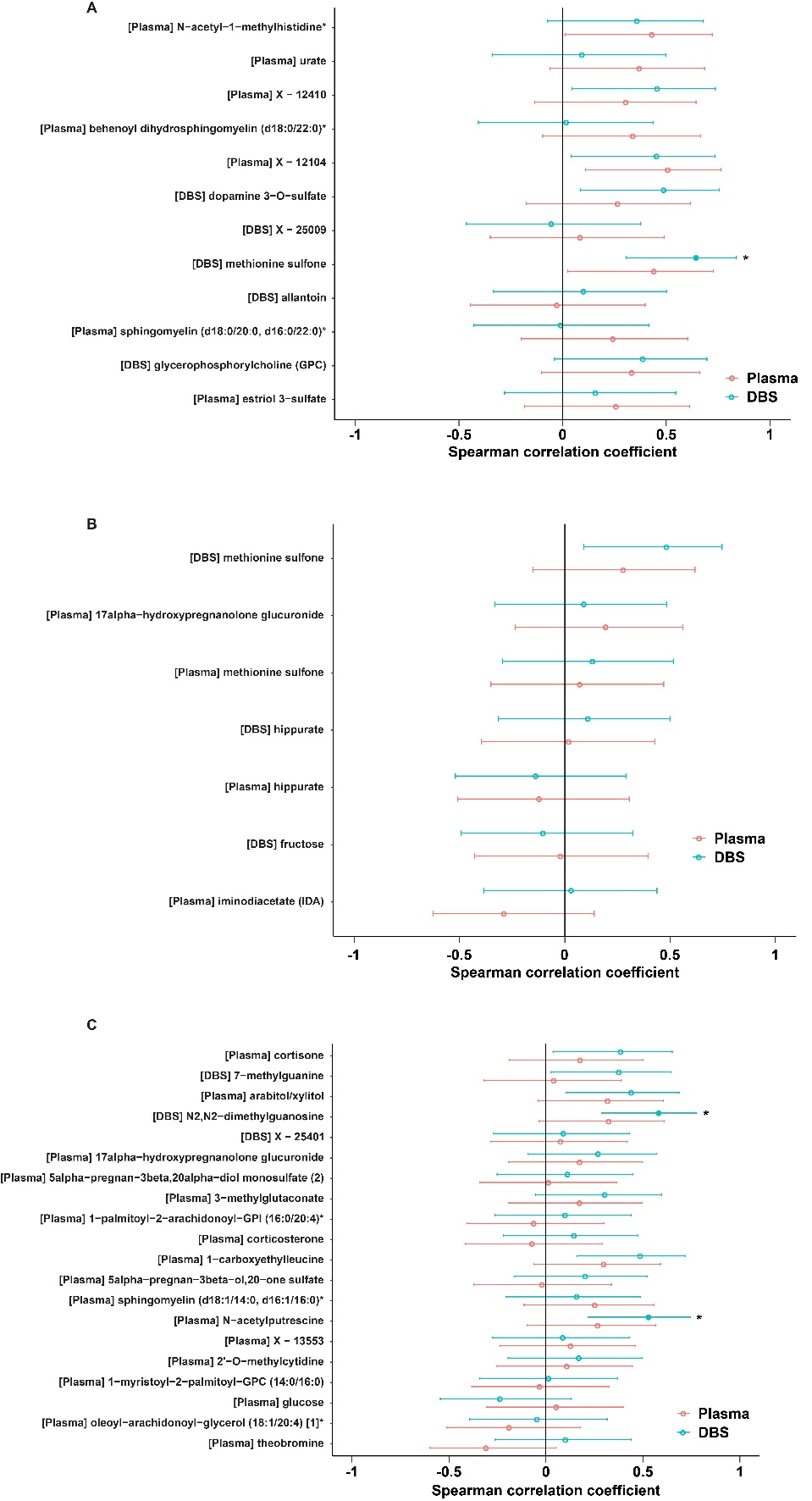


Figure S6. Correlation between levels of creatinine and features selected in random forests models of birth status in the (a) untreated, (b) zidovudine monotherapy, and (c) PI-ART groups. Spearman correlation coefficients are shown as point estimates with 95% confidence intervals, separately for plasma and DBS measurements of creatinine as indicated. Filled-in circles and corresponding asterisks denote significant correlations.


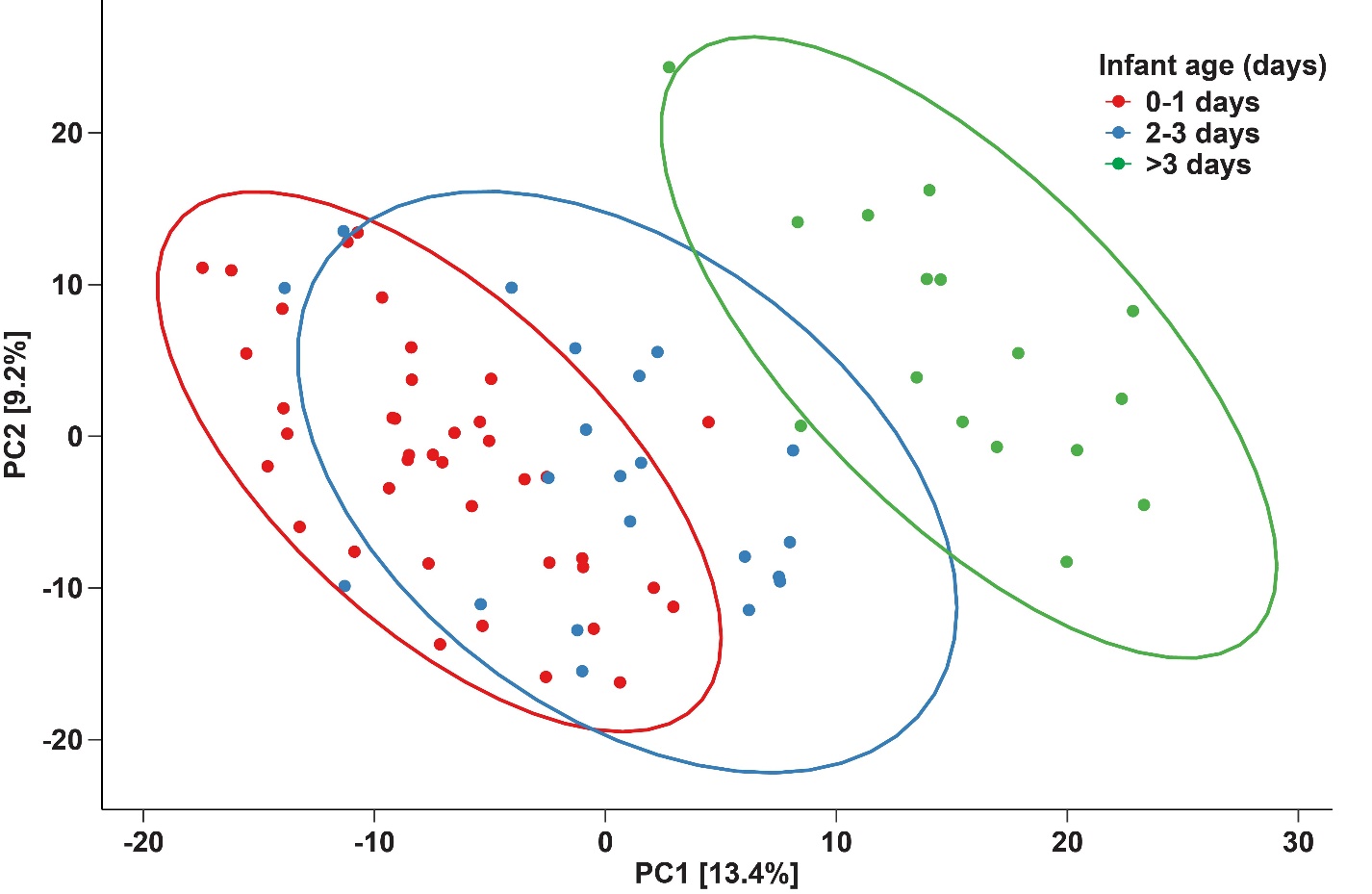


Figure S7. Principal components analysis of infant DBS metabolite profiles using Euclidean distances. Ellipses show 95% confidence areas for the age groups as marked. Numbers in brackets denote percent of overall variation explained by each component.
